# Peripheral Venous Pressure And NT-proBNP Phenotypes as Surrogates for Invasive Fontan Haemodynamics in Adults

**DOI:** 10.1016/j.cjcpc.2024.07.003

**Published:** 2024-08-02

**Authors:** William R. Miranda, C. Charles Jain, Heidi M. Connolly, Alexander Van De Bruaene, Gruschen R. Veldtman, Alexander C. Egbe

**Affiliations:** a Department of Cardiovascular Diseases, Mayo Clinic, Rochester, Minnesota, USA; b Division of Structural and Congenital Cardiology, Department of Cardiovascular Sciences, KU Leuven, Leuven, Belgium; c Scottish Adult Congenital Cardiac Service, Golden Jubilee National Hospital, Glasgow, United Kingdom

## Abstract

We hypothesized that phenotyping patients using a combination of resting peripheral venous pressure (PVP) and N-terminal pro–brain natriuretic peptide (NT-proBNP) would predict invasive Fontan haemodynamics. Accordingly, 35 adults with a history of Fontan palliation were categorized into 3 groups according to PVP and NT-proBNP values: normal, ↑NT-proBNP (≥300 pg/dL) or ↑PVP (≥15 mm Hg), and ↑PVP+↑NT-proBNP. Those in the normal values group universally had normal resting pulmonary artery wedge and Fontan pressures, with a single patient having abnormal exercise values; conversely, all patients in the ↑PVP+↑NT-proBNP group had increased resting Fontan or pulmonary artery wedge pressures, with those in the ↑NT-proBNP or ↑PVP group constituting an intermediate group.

## Résumé

Nous avons émis l’hypothèse que la description phénotypique des patients par la pression veineuse périphérique (PVP) au repos et le dosage du propeptide natriurétique de type B N-terminal (NT-proBNP) pourrait prédire le recours à l’évaluation hémodynamique invasive après l’intervention de Fontan. Trente-cinq adultes présentant des antécédents de palliation de Fontan ont été répartis dans trois groupes, en fonction de leur PVP et de leur taux de NT-proBNP : valeurs normales; ↑NT-proBNP (≥ 300 pg/dl) ou ↑PVP (≥ 15 mmHg); ↑PVP + ↑NT-proBNP. Les patients affichant des valeurs normales présentaient invariablement une pression capillaire pulmonaire bloquée (PCPB) et des pressions dans le circuit de Fontan (PF) au repos normales; un seul patient présentait des valeurs anormales à l’effort. À l’inverse, tous les patients du groupe ↑PVP + ↑NT-proBNP présentaient des valeurs élevées de PCPB ou de PF au repos; les patients du groupe ↑NT-proBNP ou ↑PVP formaient un groupe intermédiaire.

Systemic venous hypertension is an inevitable consequence of the Fontan procedure, and the degree of elevation in central venous pressure has been consistently shown to be an independent predictor of outcomes in this population. Despite this critical role, assessing Fontan pressures (FP) in routine patient evaluation continues to pose a challenge to the clinician. Accordingly, some practices pursue periodic cardiac catheterization in the follow-up of Fontan patients. However, this approach carries inherent risk and cost. Peripheral venous pressure (PVP) measurement has emerged as an accurate tool in the noninvasive evaluation of FP.^1^^,^^2^ Separately, we have reported that N-terminal pro–brain natriuretic peptide (NT-proBNP) levels ≥300 pg/dL were associated with unfavourable exercise invasive Fontan haemodynamics.^3^ Accordingly, we hypothesize that phenotyping patients using a combination of resting PVP and NT-proBNP data would predict invasive Fontan haemodynamics among adults and further refine the information provided by PVP alone.

The study is a retrospective analysis of 35 consecutive adults (aged ≥18 years) with a history of Fontan palliation with PVP measured during cardiac catheterization and available NT-proBNP data. As previously stated,^1^ following the publication of Tan et al.,^2^ our laboratory began to routinely measure PVP at cardiac catheterization of Fontan patients to allow for subsequent serial measurements during their outpatient and inpatient care. The Institutional Review Board approved the study, and only patients providing authorization for the use of their records for research purposes were included.

Cardiac catheterization was performed in the supine position, in a fasting state under mild sedation. At the time of the procedure, PVP was measured via a 3-way stopcock connected to an 18-20G intravenous line previously placed in the upper extremity for administration of periprocedural medications. The arm was allowed to rest comfortably on the examination table, parallel to the body. Pressure zeroing was performed in a standard fashion at the mid-chest level. PVP values reported herein were measured simultaneously with superior vena cava (SVC) pressures in all but one patient.

Elevated (↑) PVP was defined as a resting value ≥15 mm Hg, which corresponds to an FP ≥14 mm Hg.^1^ ↑NT-proBNP level was defined as ≥300 pg/dL.^3^ Patients were subsequently categorized into 3 groups according to PVP and NT-proBNP values: normal, ↑NT-proBNP or ↑PVP, and ↑PVP+↑NT-proBNP. Increased resting FP and pulmonary artery wedge pressure (PAWP) were defined as ≥14^4^ and ≥12 mm Hg,^5^ whereas increased exercise values were defined as >25 and >20 mm Hg,^6^ respectively. In agreement with previously used methodology,^6^ FP corresponds to pulmonary artery pressures.

Continuous data are presented as medians (25^th^-75^th^ percentile), whereas categorical data are presented as counts (%). Between-group differences in haemodynamics were assessed using the Fisher exact test or the Wilcoxon test. The comparison between SVC and PVP was performed by simple linear regression. A *P* value of <0.05 was considered statistically significant.

Underlying congenital lesions were tricuspid atresia in 8 patients (23%), hypoplastic left heart syndrome in 8 (23%), double inlet ventricle in 7 (20%), double outlet right ventricle in 5 (14%), and other in 7 (20%). Fontan connections were extracardiac conduit in 18 (51%), lateral tunnel in 10 (29%), atriopulmonary in 5 (14%), and Kawashima-type and intra-atrial in 1 each (3%). None of the patients had SVC obstruction. The correlation coefficient between PVP and simultaneous SVC measurements was 0.92 (PVP-SVC pressure difference 1 [0; 2] mm Hg).

**Figure 1:**
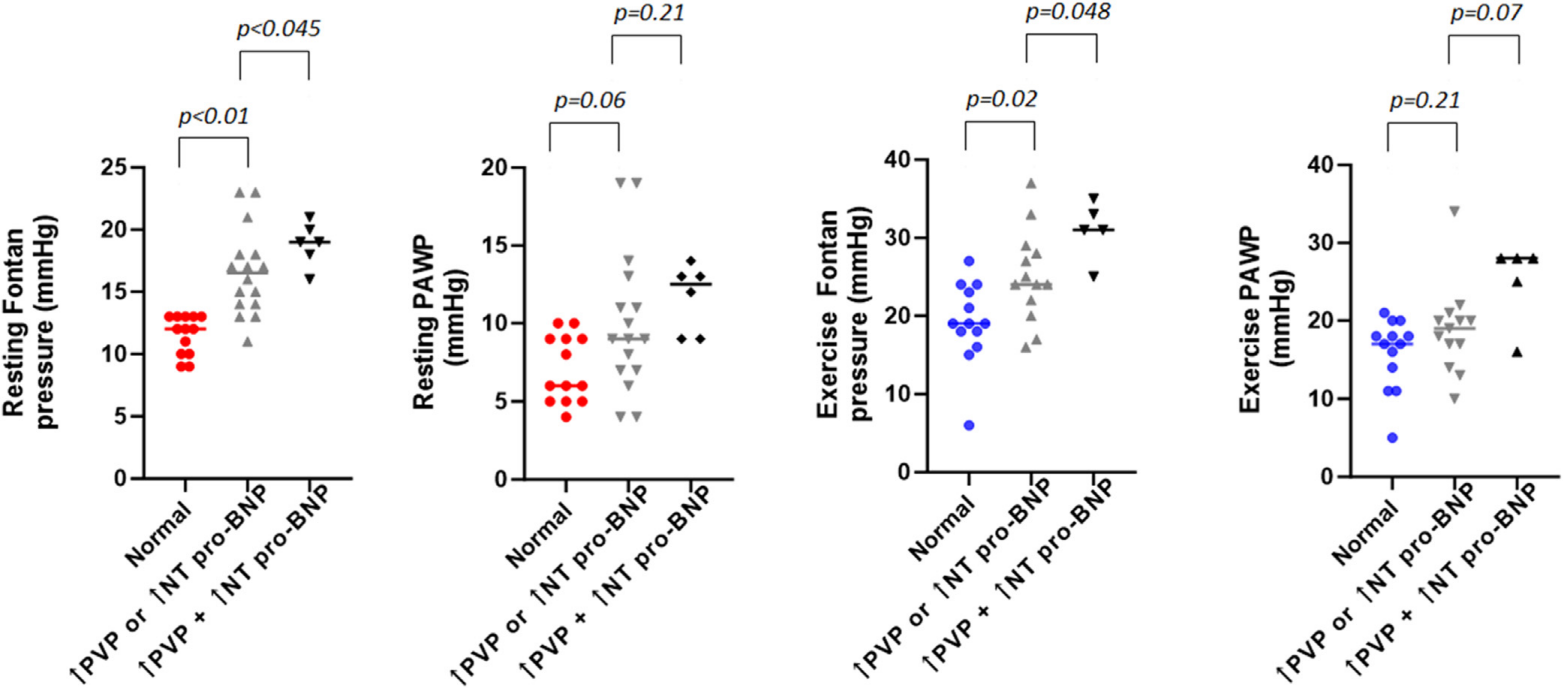
Resting and exercise haemodynamics according to PVP and NT-proBNP phenotypes. NT-proBNP, N-terminal pro–brain natriuretic peptide; PAWP, pulmonary artery wedge pressure; PVP, peripheral venous pressure.

Clinical and haemodynamic data for the individual phenotypes are presented in Table 1 and Figure 1. The distribution according to PVP-biomarker phenotypes was as follows: normal in 13 patients (38%), ↑NT-proBNP or ↑PVP in 16 (46%), and ↑PVP+↑NT-proBNP in 6 (18%). Median FP numerically increased across these phenotypes, with FP being significantly lower in the normal group than in the ↑NT-proBNP or ↑PVP group (*P* < 0.01) and lower in the latter than in the ↑PVP+↑NT-proBNP group (*P* = 0.045). There was a trend towards PAWP being lower in the normal group than in the ↑NT-proBNP or ↑PVP group (*P* = 0.06), whereas no significant differences were seen between the ↑NT-proBNP or ↑PVP and ↑NT-proBNP+↑PVP groups. Noteworthily, none of the patients in the normal phenotype had increased resting FP or PAWP. In contrast, increased FP was present in all patients in the ↑PVP+↑NT-proBNP group, with 67% of them having an increased PAWP.

**Table 1:** Clinical characteristics

Characteristic	Normal (n = 13)	↑NT-proBNP or ↑PVP (n = 16)	↑PVP+↑NT-proBNP (n = 6)
Age (y)	31 (25; 38)	31 (23; 37)	38 (34; 48)†
Female sex	6 (47)	6 (38)	2 (33)
Body mass index (kg/m^2^)	24 (22; 26)	27 (21; 34)	27 (25; 28)
Patent fenestration	1 (8)	4 (25)	1 (17)
Right ventricular morphology	6 (46)	8 (50)	2 (33)
Atriopulmonary Fontan	0	4 (25)	1 (17)
NYHA class III-IV	3 (23)	6 (38)	2 (40)
History of atrial arrhythmias	3 (23)	6 (38)	6 (100)†
Creatinine clearance <60 (mL/min)	0	2 (13)	1 (17)
Medications			
Diuretics	1 (8)	9 (57)∗	6 (100)
β–Blocker	5 (38)	3 (19)	3 (50)
ACEi/ARB	8 (62)	10 (63)	1 (17)
Aldosterone antagonist	1 (8)	7 (44)∗	3 (50)
Phosphodiesterase type 5 inhibitor	4 (31)	4 (25)	2 (33)
Laboratory/imaging			
NT-proBNP (pg/mL)	112 (73.5; 163)	230 (159; 453)∗	442 (317; 927)
MELD-XI	9.4 (9.4; 11.4)	10.8 (9.4; 14.1)	11.9 (9.4; 19.2)
Spleen size (cm)	12 (11.3; 13.2)	12.7 (12.3; 13.9)	15.3 (13.4; 17.4)†
Echocardiography			
Ejection fraction (%)	53 (44; 56)	55 (46; 58)	55 (49; 59)
≥Moderate AV regurgitation	1 (8)	5 (31)	1 (17)
Noninvasive cardiopulmonary exercise test (n = 25)			
Peak VO_2_ (mL/kg/min)	22.9 (16.4; 24.7)	18.7 (16.2; 20.5)	14.4 (11.9; 19.5)
Peak VO_2_ (% of predicted)	61 (49; 71)	47 (44; 58)	50 (34; 56)
Resting cardiac catheterization			
PVP (mm Hg)	12 (10; 13)	16.5 (14; 18)∗	19 (17.5; 20.3)
Fontan (mm Hg)	11 (10; 12)	14 (11; 16)∗	18 (17; 20)†
PAWP (mm Hg)	6 (5; 9)	9 (7; 13)	13 (9; 13)
Arterial O_2_ saturation (%)	95 (93; 97)	91 (86; 94)∗	92 (77; 94)
Cardiac index (L/min/m^2^)	2.6 (2.3; 3.1)	2.2 (1.7; 2.5)	2.1 (1.5; 2.6)
PVRi (U.m^2^)	1.2 (0.8; 2.4)	1.8 (1.2; 2.8)	2.6 (2.2; 3.3)
Fontan ≥14 (mm Hg)	0	11 (68)∗	6 (100)
PAWP ≥12 (mm Hg)	0	4 (25)	4 (67)
Exercise cardiac catheterization (n = 31)			
Fontan (mm Hg)	19 (17; 24)	24 (21; 29)∗	31 (28; 34)†
PAWP (mm Hg)	17 (13; 19)	19 (16; 21)	28 (21; 28)
Fontan ≥25 (mm Hg)∗	1 (8)	5 (38)	4 (80)
PAWP ≥20 (mm Hg)	1 (8)	3 (23)	4 (80)†

Data are presented as median (25th-75th percentile) and n (%).

ACEi/ARB, angiotensin-converting enzyme inhibitor/angiotensin receptor blocker; AV, atrioventricular valve; MELD-XI, Model for End-Stage Liver Disease Excluding (INR); NT-proBNP, N-terminal pro–brain natriuretic peptide; NYHA, New York Heart Association; PAWP, pulmonary artery wedge pressure; PVP, peripheral venous pressure; PVRi, pulmonary vascular resistance index; SVC, superior vena cava; VO_2_, oxygen consumption.

∗ *P* value <0.05 for comparisons between the normal and ↑NT-proBNP or ↑PVP groups.

† *P* value <0.05 for comparisons between the ↑NT-proBNP or ↑PVP and ↑NT-proBNP+↑PVP groups.

Exercise catheterization^7^ was pursued in 31 patients. Exercise FP were lower in the normal group than in patients in the ↑NT-proBNP or ↑PVP group (*P* = 0.02), whereas those in the ↑PVP+↑NT-proBNP group had higher values than those in the ↑NT-proBNP or ↑PVP group (*P* = 0.048). An exercise FP >25 mm Hg and a PAWP >20 mm Hg were seen in a single patient (8%) in the normal group. Conversely, this was present in 80% of individuals in the ↑PVP+↑NT-proBNP group.

The modest between-group differences shown in Table 1 illustrate the challenges in estimating Fontan haemodynamics based on clinical information alone. The current findings suggest that a combination of resting PVP and NT-proBNP levels could be used to phenotype adults after Fontan and predict their haemodynamics. Importantly, those with normal PVP and biomarker levels universally had normal resting PAWP and FP, with a single patient having abnormal exercise values. In the absence of another indication for catheterization such as Fontan pathway obstruction, formal cardiac catheterization in this phenotype might have low yield. In contrast, all patients in the ↑PVP+↑NT-proBNP group had increased resting FP or PAWP, thus providing physiological underpinnings for their functional capacity or higher comorbidity burden (atrial arrhythmias, abnormal liver testing, and splenomegaly). Finally, those in the ↑NT-proBNP or ↑PVP group constitute an intermediate group, who could benefit for a more in-depth invasive haemodynamic evaluation.

We acknowledge the limitations of the study, including the retrospective and heterogeneous nature of the study population. Because of the small sample, we were unable to analyse isolated increases in PVP or NT-proBNP as separate groups. Lastly, the NT-proBNP cutoff used here was arbitrary,^3^ and the optimal level to predict abnormal Fontan haemodynamics remains to be determined.

We hope that our current findings stimulate subsequent studies on phenotyping adult Fontan patients using PVP and serum biomarkers. If replicated, our observations would support the routine use of PVP in outpatient care of patients after Fontan palliation, permitting early recognition of untoward underlying haemodynamics, individualizing the need for cardiac catheterization, and minimizing invasive procedures in some.
